# Recent Advances in Generation and Detection of Orbital Angular Momentum Optical Beams—A Review

**DOI:** 10.3390/s21154988

**Published:** 2021-07-22

**Authors:** Denis M. Fatkhiev, Muhammad A. Butt, Elizaveta P. Grakhova, Ruslan V. Kutluyarov, Ivan V. Stepanov, Nikolay L. Kazanskiy, Svetlana N. Khonina, Vladimir S. Lyubopytov, Albert K. Sultanov

**Affiliations:** 1 Telecommunication Systems Department, Ufa State Aviation Technical University, 450008 Ufa, Russia; grakhova.ep@ugatu.su (E.P.G.); kutluyarov.rv@ugatu.su (R.V.K.); stepanov.iv@ugatu.su (I.V.S.); sultanov.ah@ugatu.su (A.K.S.); 2 Department of Technical Cybernetics, Samara National Research University, 443086 Samara, Russia; butt.m@ssau.ru (M.A.B.); kazanskiy@ipsiras.ru (N.L.K.); khonina@ipsiras.ru (S.N.K.); 3 Institute of Microelectronics and Optoelectronics, Warsaw University of Technology, 00-662 Warszawa, Poland; 4 Image Processing Systems Institute Branch of the Federal Scientific Research Center “Crystallography and Photonics” of Russian Academy of Sciences, 443001 Samara, Russia; 5 Center for Photonics and Quantum Materials, Skolkovo Institute of Science and Technology, 121205 Moscow, Russia; v.lyubopytov@skoltech.ru

**Keywords:** orbital angular momentum, optical waveguide, diffractive optic elements, spatial light modulator, metasurfaces, computer-generated holograms

## Abstract

Herein, we have discussed three major methods which have been generally employed for the generation of optical beams with orbital angular momentum (OAM). These methods include the practice of diffractive optics elements (DOEs), metasurfaces (MSs), and photonic integrated circuits (PICs) for the production of in-plane and out-of-plane OAM. This topic has been significantly evolved as a result; these three methods have been further implemented efficiently by different novel approaches which are discussed as well. Furthermore, development in the OAM detection techniques has also been presented. We have tried our best to bring novel and up-to-date information to the readers on this interesting and widely investigated topic.

## 1. Introduction

Optical beams transporting orbital angular momentum (hereafter expressed as OAM), also known as optical vortices (OVs), have piqued researchers’ interest in recent years due to their unique properties [1]. A helical wavefront and a phase proportional to exp(*ilϕ*) describe such beams, where *l* is an integer known as the topological charge, which could take any value. Optical OAM modes form an orthogonal basis that can be utilized to fundamentally enhance the capability of fiber optic [2], free-space, and quantum communications [3]. Also, the phase of optical vortices is utilized in the analysis of the laser field’s polarizing properties. For example, a single fork-shaped phase grating matched with first-order vortices for detecting radially and azimuthally polarized laser beams [4], and more complex multi-order optical elements matched with vortices of different orders, make it possible to uniquely determine various combinations of the cylindrical polarization state and the vortex phase of the laser beam [5,6,7]. Optical tweezers and spanners [8,9,10,11,12], non-diverging speckles [13], imagining and microscopy [14,15,16,17], novel sensing technologies for detecting molecules and nanostructures [18,19,20,21,22], and object motion detection [23,24] are only a few of the applications for optical vortices that have been proposed so far. As a result, there is a growing demand for efficient beam generation methods.

Since Allen and coworkers’ seminal paper [25], light beams carrying OAM have spawned a burgeoning area of study, resulting in a plethora of studies and applications [26], including particle trapping [27], tweezing and manipulation [28], astronomical coronagraphy [29], mode-division multiplexing [30], and security [31]. Discrete optical elements perform most methods of generating optical vortices, which are traditionally divided into two groups: spatial and fiber generating methods [32]. Diffractive optical components [33,34], cylindrical lens mode converters [35], spiral phase plates (SPPs) [36,37], circular and spiral gratings [38], spatial light modulators [39], metamaterials [40], and q-plates [41] are among the devices in the first category. On the millimeter scale, the second category of fiber-based systems, such as fiber gratings [42], chirally coupled core fibers [43], and photonic lanterns [44], seem to provide more promising solutions in terms of system size. In addition, the solutions based on photonic integrated circuits (PICs), due to their energy efficiency, small footprint, and performance reliability, become a sensible option for implementing the OAM-based applications. Furthermore, PIC’s fabrication processes ensure repeatability in the large-scale production of the developed devices. The PIC-based OAM modes generator satisfies the modern trend toward optical integration in terms of practical miniaturization and multiplexing optical signals for transmission in a single propagation medium.

**Figure 1:**
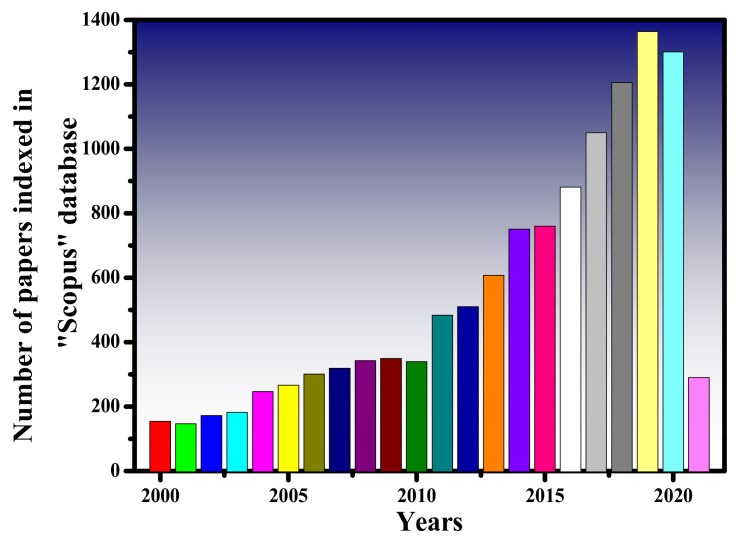
The number of papers related to the “Orbital angular momentum” topic searched in the Scopus database for the years 2000–2021.

The generation of in-plane or out-of-plane light with OAM is an interesting topic that has been studied by researchers all over the world. The popularity of this topic can be witnessed in Figure 1 where the number of published papers index in the Scopus database is presented from the years 2000–2021. It shows a linear trend in the publication of papers as the topic is developing with time and several new techniques have been proposed for the efficient generation of light with OAM.

In this paper, the most significant and recent advances in the generation of light with OAM are discussed. We have focused on three different methods that are popular among the scientific community and widely researched in recent times. In Section 2, OAM generation via diffractive optics is discussed. It involves three significant methods, i.e., spiral phase plate (hereafter expressed as SPP), computer-generated holograms (hereafter expressed as CGHs), and diffractive optical elements (DOEs) that are considered in detail. Section 3 is devoted to metasurfaces (hereafter expressed as MSs). In recent times, the adaptable MSs, 2D arrays of subwavelength structures with space-variant phase responses, deliver robust and accessible methods for the generation of OAM. Section 4 discusses recent developments in OAM generation in photonic integrated circuits (PICs). These OAM generators can be separated into two categories; one is that OAM beams are created out-of-plane and transmitted in free space, while the other is that OAM beams are produced in-plane and propagated in waveguides (hereafter represented as WG). The latter has attracted a lot of interest in fully utilizing the distinctive characteristic of OAM beams in guided optics. Nevertheless, to ensure uniform spatial distributions of multi-coupled WGs, the majority of the integrated OAM generators need extremely critical dimension control technology. In the end, a brief overview of the OAM detection methods is discussed in Section 5 followed by the conclusion in Section 6.

Allowing a light beam to transmit through a medium with spiral inhomogeneity in the longitudinal path to produce an integer phase stage along the azimuthal angle is the most explicit method to establish a helical wavefront. The spiral phase plate (SPP), which was first used in 1994, is a straightforward route to assemble a plate with a helical surface [36]. Usually, the helical surface is discretized into various steps as diffractive elements to make the fabrication process simpler. The working wavelength can be slightly tuned by matching the refractive index. Another approach for creating optical vortices is to utilize a computer-generated hologram (CGH) [45,46]. A spiral interfering fringe is produced when an OV beam bearing a spiral wavefront collinearly interferes with a Gaussian beam, with the number and rotating direction of the spiralling arms signifying the number and sign of the topological charge, respectively. As it hits with an off-axis plane wave, it produces a forked grating with a visible defect on the fringes where a phase singularity occurs. These distinctive interference patterns were also commonly used to describe an OV beam’s topological charge. The spiral fringe or the forked grating may be used to create a hologram plate established on the interference property. Commercial spatial light modulators (SLMs) established on pixelate liquid crystals can be programmed to produce holograms through a consumer terminal, and are widely used in this way [47].

Recent advancements in flexible MSs have also accelerated the development of efficient and convenient OAM generation routes [48,49,50,51,52,53]. MS is based on frequency selective surfaces (FSSs) [54]. They are made up of different shaped and oriented subwavelength scatterers [55]. As a result of the high feasibility of tailoring the geometry and orientation of the ultracompact scatterers, they go beyond traditional FSSs. The sudden phase shift at the scatterers causes MSs to change the wave properties locally. Scatterers can cover an entire 2π phase shift by adjusting the geometry or alignment, allowing for arbitrary beam formation [56]. In the meantime, scatterers can be built to adjust the wave front. They may also have a magnetic response in addition to the electric response. Nonperiodic MSs that respond to both electric and magnetic fields have been introduced. These surfaces are referred to as metamaterial Huygens’ surfaces, since their design is based on a rigorous interpretation of Huygens’ principle [57]. The magnetic response aids in compensating for the impedance mismatch at the MS interface, allowing for perfect performance. The design principles for OAM generation lead to one familiar rule: the implementation of the azimuthal phase term e*^ilϕ^*to EM waves, even though there are numerous models in various categories and appropriate for different application circumstances. They are divided into two groups: those that are independent of wave polarization and those that are dependent on it.

**Figure 2:**
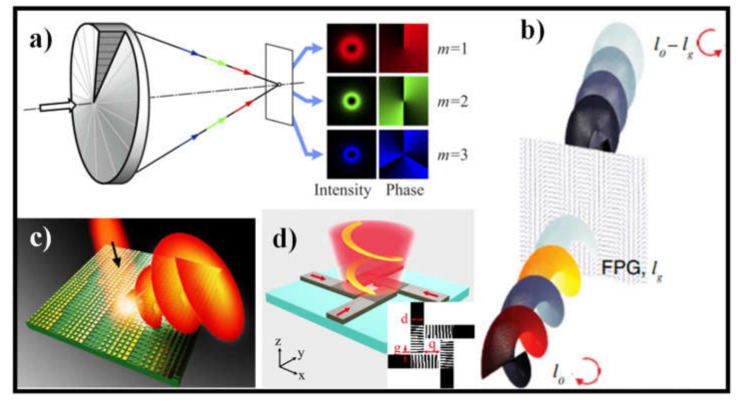
Methods to generate light with OAM: (**a**) Shift in the OAM order by illuminating a 3D element with the laser light of different wavelengths [58]; (**b**) OAM generator via CGH [59]; (**c**) Schematic of the OV beam generator from MS [60]; and (**d**) The schematic illustration of the OV beam generator on the embedded multi-WG [61].

Figure 2 demonstrates four different methods used for the creation of light with OAM which are widely used among the researchers [58,59,60,61]. Another device, comprising a mutable amplitude splitter (VAS) and an OAM emitter, has recently been suggested, in which the comparative amplitude of the two OAM states with inverse chirality can be tuned [62]. Furthermore, many methods such as WG-array centered radial grating coupling system [63], hybrid 3D PIC device [64], organic semiconductor μ-disk laser, and micro-scale SPP integrated VSCEL [65] have demonstrated readily OAM generation and (de-)multiplexing (multiple OAM states superposition) functions, which differ from this azimuthal grating coupling system. These devices demonstrated the possibility of developing advanced OAM functions on scalable and dense PICs using on-chip dynamical control of OAM states [66].

## 2. OAM Emitters Based on Diffractive Optic Elements

Fiber optics are now employed in the backbone part of modern data communications. However, for specific applications, fiber is not practical due to the immobility of the sending and receiving ports connected to the fiber laid down. Free space optical networking, also identified as wireless optical communication, reduces the system’s spatial dependency. Furthermore, applying OAM to a beam of light mitigates some of the shortcomings that would otherwise be present. A significant limiting factor on signal path length is the rate at which light signals spread through the air. An image has been effectively sent 3 km through the air utilizing OAM, despite considerable turbulence along the path length [67].

Since laser beams with OAM are of special interest for data channel multiplexing [2,68,69]. Many studies are devoted to the propagation of OV beams as in free space [70,71,72,73] and in optical fibers [74,75,76]. To implement multichannel information transfer using OAM, a certain superposition of OVs with a given energy and phase distribution in the transmitter must be excited on the transmitting side, so each mode transmits an independent information signal. This can be done using elements of diffractive optics. The design doctrines for OAM production leads to one general rule: the implementation of the azimuthal phase term e*^ilϕ^* to EM waves, even though there are numerous models in different types and appropriate for distinct application circumstances. The first scheme makes use of isotropic materials like SPPs [77] and CGHs [78]. An SPP is the simplest device for manipulating OAM modes at millimeter frequencies. An SPP is a dielectric slab of material with an azimuthally reliant on thickness that causes incident radiation to undergo an azimuthal phase change [79,80]. The SPP’s total step height, *h*, is selected so that the total phase change around the SPP’s center is an integer multiple of 2π*l*, where *l* is an integer. The azimuthal mode number *l* of the incident radiation is modified because of this, such that: $\Delta l=\frac{h\Delta n}{\lambda }$, where Δ*n* is the refractive index difference between the dielectric material and the surrounding medium, and *λ* is the incident wavelength.

**Figure 3:**
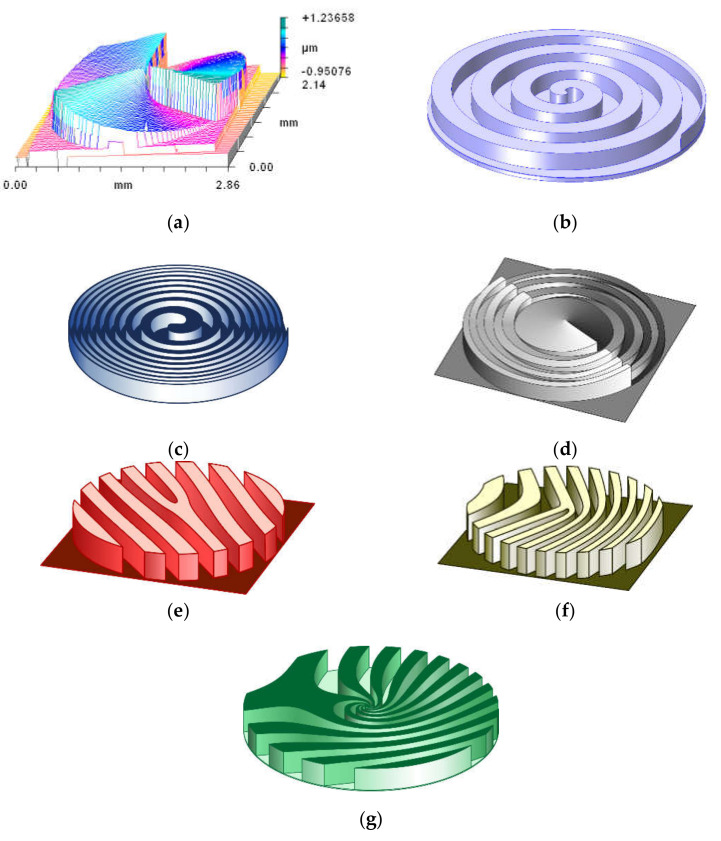
Elements of singular optics: (**a**) SPP. Reprinted with permission from ref. [36]. Copyright 1994, Elsevier; (**b**) spiral axicon; (**c**) spiral zone plate; (**d**) OV autofocusing optical element; (**e**) fork grating; (**f**) curved fork grating; and (**g**) grating for OV hyper-geometric laser beams generation.

In addition to SPPs, various DOEs (see Figure 3) are actively used to generate OVs, such as spiral axicons [81,82,83], generating Bessel beams of high orders, and spiral zone plates [84,85,86] which are a combination of a focusing lens and an SPP. Recently, unconventional focusing elements have been used to provide abruptly autofocusing [87,88,89]. The listed elements form OV beams on the optical axis. To generate OV beams at an angle to the optical axis or in different diffraction orders, fork gratings are used [90,91,92,93], as well as curved fork gratings [94,95,96]. Regular fork gratings correspond to plane wave multiplexing, while curved fork gratings provide conical wave multiplexing. More complex elements are also known, for example, for multiplexing Airy beams [97], hypergeometric beams [98], and Laguerre-Gauss modes [99,100,101].

DOEs are relatively simple optical elements, as shown in Figure 3. To generate a co-axial linear arrangement of a finite number of OV modes with given weights (or information codes) more complex optical elements are used [102,103,104,105], which are calculated using various coding methods [106,107,108,109]. Note, the classical diffraction SPPs are manufactured for a specific laser wavelength. However, refractive (with high relief) SPPs have been successfully used for different wavelengths [110,111] allowing the OVs generation of various orders, including fractional orders [58]. The advantages of SPPs made from solid materials are high damage threshold and efficiency.

**Figure 4:**
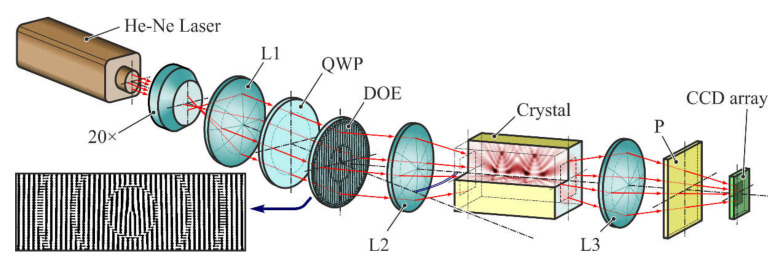
Optical system for generation of vector OV beams by a combination of DOEs and anisotropic crystals: a He-Ne laser emitting linearly polarized light which was expanded using an objective (L1), a quarter-wave plate (QWP) was utilized to alter the linearly polarized beam into the circularly polarized beam, the diffractive optical element (DOE) generates OV Laguerre-Gaussian modes which are focusing by a lens (L2) into a c-cut CaCO_3_ crystal, cylindrically polarized beams (with radial or azimuthal polarization) are imaging by a lens (L3) at CCD array and analyzed by a polarizer (P). Reprinted with permission from ref. [115]. Copyright 2017, Elsevier.

The same qualities are inherent in devices based on solid anisotropic crystals, which are often used not only to generate OV beams but also cylindrically polarized beams [112,113,114,115,116]. For example, Figure 4 shows an optical system for the generation of OV Laguerre-Gaussian beams with radial or azimuthal polarization by a combination of DOEs and anisotropic crystals [115]. Spatial light modulators (SLMs) are very convenient and versatile devices for the formation of vector OV beams [117,118,119,120,121]. However, it is known that SLMs are characterized by low efficiency, resolution, and when used with high-power lasers they require additional cooling systems [122]. Thus, the application of DOEs remains relevant.

## 3. MSs for Generation of Vector OV Beams

Alternatively, an antenna or antenna array’s excitation may be explicitly modified to fulfill the necessary phase requirement, allowing an OAM wave to be emitted out [123]. The abrupt phase change at scatterers on an MS can also be used to create e*^ilϕ^*. The resonant frequency of the scatterer is modified by changing its geometry, causing the phase shift to differ at the desired frequency. After optimization, a total of 2π phase shift is achieved, and the effective generation of various OAM states has been demonstrated [124]. Tunable scatterers stocked by varactor diodes have recently been suggested as a convenient way to generate multiple OAM modes [125]. Scatterers can also be rendered anisotropic to regulate various polarizations independently. Even if the scatterer’s responses are polarization-dependent, the helicity of the emitted OAM is independent of the incident wave’s polarization state. In other words, the helicity is fixed.

**Figure 5:**
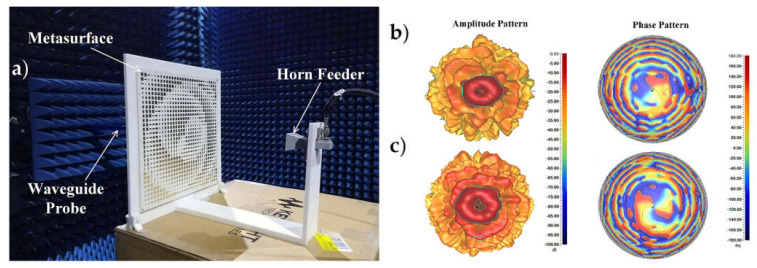
(**a**) The fabricated MS and its experimental setup with a WG probe in the near field chamber [126]. Radiation forms of the MS: (**b**) x-polarization, OAM mode *l_x_* = +1 [126]; and (**c**) y-polarization, OAM mode *l_y_* = −2 [126].

To generate dual-mode convergent OAM beams, a high-efficiency dual-polarized transmissive MS is presented in [126]. To achieve the phase modulation for the orthogonal polarizations, the recommended transmissive MS consists of dual-polarized MS units made up of four packed stripes coupled through a Jerusalem-cross aperture on the interlayer ground. The MS units are capable of absolute 2π phase modulation with low transmission loss. The suggested transmissive MS for both orthogonal polarizations, which has been proven numerically and experimentally, stimulates converged OAM beams with mode numbers *ℓ_x_* = +1 and *ℓ_y_* = −2. The MS prototype with 30 × 30 units was fabricated and tested to authenticate the efficacy of the engineered MS for generating dual-polarized dual-mode converged OAM beams. The measurements are carried out in the near-field anechoic chamber using a 3D platform and an open-ended WG probe, as shown in Figure 5a [126]. Figure 5b,c depicts the measured full hemisphere far-field radiation patterns in magnitude and phase for the two orthogonal polarized OAM modes, with the excessive intensity annular tapered patterns, as well as the typical spiral phase fronts and on-axis phase singularities, obviously visible [126].

The transformation and coupling of SAM and OAM are the basis for the second scheme [127,128]. This occurs in media that are nonuniform and anisotropic, such as q-plates [129]. Q-plates have a spatially shifting optical axis and change the circular polarization state from left to right or right to left. AM conservation law may be used to justify their behavior. SAM changes by a factor of ±2ħ when the circular polarization state is flipped. The cumulative AM is conserved when the q-plate is cylindrically symmetrical so that the output wave must have an OAM of ∓2ħ. When the q-plate is not cylindrically symmetrical, it introduces extra AM into the device, allowing for the generation of different orders of OAM in the output wave. Because of their versatility and anisotropic properties, liquid crystals are commonly used to make q-plates. The birefringent retardation in liquid crystals varies with temperature on external voltage, allowing the conversion efficiency to be tweaked [130]. When the retardation is π, optimum productivity is achieved. The function of q-plates has been implemented using MSs [78,131,132]. The rotation of the optical axis of a q-plate, along with the insertion of a geometric step, is analogous to the flipping of the circular polarization state accomplished by anisotropic scatterers and the rotation of the scatterers. Geometric phase MSs is the name given to this type of MS [133]. Geometric-phase MSs, unlike MSs that use the first scheme, generate OAM in conjunction with a transition in the polarization state of the output wave, which is radically different from the first scheme. The incident SAM determines the handedness of the generated OAM.

Of course, MSs are very promising devices for generating vector OV beams, since they are compact optical elements and can simultaneously perform several operations: to not only generate OV phase and perform polarization conversion, but also focus or multiplexing beams. There are metal MSs (subwavelength structures etched on metal films) [134,135] and dielectric MSs [136,137]. Metal subwavelength gratings operate as a rule in a reflecting mode and are less chemically resilient to an assertive medium. The main disadvantage of subwavelength dielectric gratings is the nonuniformity of amplitude transmission for different polarizations. This drawback can be prevented by merging several functions: polarization transformation by subwavelength gratings and focusing, for orthogonal ridges in adjacent Fresnel zones [138,139].

Large numbers of OAM modes are normally controlled with a diffractive hologram projected on an SLM in optical systems, however, due to the comparatively long wavelengths, comparable systems do not occur for millimeter and radio frequencies. The results of measurements performed on a modular SPP design capable of producing millimeter-wavelength beams with an azimuthal mode number of *l* ± 10 is offered [140]. The plate is made up of 10 single modules that interlock to form the entire plate assembly, allowing for better machining precision than traditional methods. As a result, this design may be utilized in millimeter-wavelength systems that involve massive OAM mode manipulation. The plate was made of polypropylene and tested at a frequency of 100 GHz. Three near field surfaces behind the plate were measured using a 3D field scanner. The OAM intensity ring was visible in intensity measurements, and phase measurements revealed 10 phase dislocations, indicating appropriate functionality [140].

**Figure 6:**
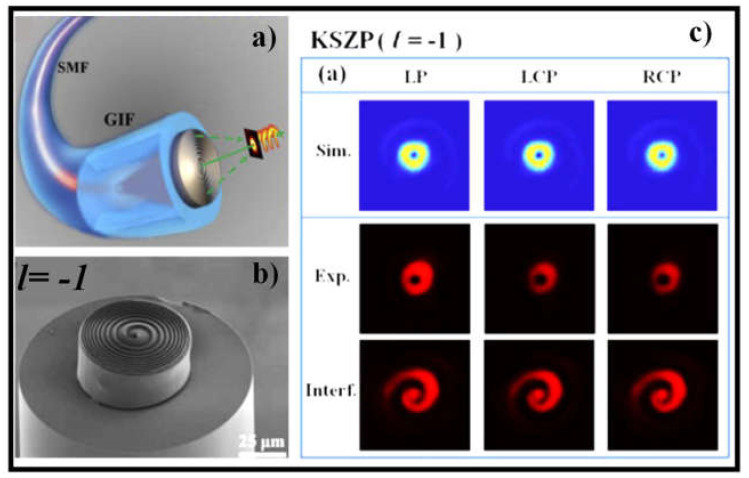
(**a**) Graphical design of all-fiber focused OV beam generator [141], (**b**) SEM image of the nanoimprinted KSZP microstructure with topological charge *l* = −1 [141], and (**c**) focal spot profiles acquired from FDTD simulation (top row), experimental measurement (middle row), and the measured coaxial interference forms (bottom row) [141].

In [141], an all-fiber high-efficiency OV beam generator is demonstrated experimentally. The generator is created by combining a kinoform spiral zone plate (KSZP) on top of the composite fiber structure, as illustrated in Figure 6a, using fs-laser two-photon polymerization 3D nanoimprinting. By overlaying a spiral phase onto a kinoform lens, the KSZP with spiral continuous-surface relief function is created, allowing an all-incident beam to be efficiently concentrated and transformed into a single-focus OV beam without the unwanted zero-order diffracted light and extra high-order focus. The SEM image of the nanoimprinted KSZP microstructure with topological charge *l* = −1 is shown in Figure 6b. Experiments show that the all-fiber generators’ focusing efficiency and OV purity are over 60% and 86%, respectively, under arbitrary polarized light incident conditions, which is significantly greater than a typical binary SZP integrated on an optical fiber facet [141]. The simulated and experimental focal spot profiles along with the coaxial interference patterns are shown in Figure 6c. The suggested all-fiber photonic devices have encouraging capacity in optical communication, particle manipulation, and quantum computing applications because of their compact size, versatile nature, polarization insensitivity, superior focusing power, and high OV purity.

## 4. OAM Beams Generation with Photonic Integrated Circuits

**Figure 7:**
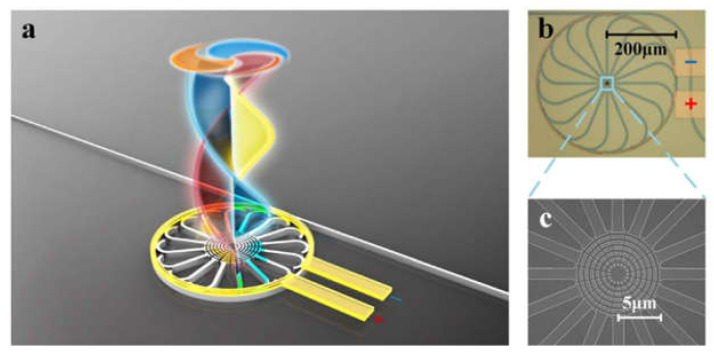
The integrated photonic emitter: (**a**) The graphical illustration [145]; (**b**) the micrograph [145]; and (**c**) the SEM image of the manufactured device [145].

There are two types of PIC solutions for generation and (de)multiplexing of OAM-beams currently available: out-of-plane and in-plane solutions. The out-of-plane approach implies the principles of scattered field generation with a help of single passive PIC elements or integrated with a light source. Most methods for out-of-plane generation and (de)multiplexing demonstrate good potential for scalability. For example, a CMOS-compatible PIC for generating or receiving azimuthally or radially polarized beams is revealed in [63,142], which is based on the SOI platform. The PIC uses annular gratings and a star coupler and can focus the beam on its own, so no external lenses are needed. It was validated, that the device is mode-number scalable, which is obtained by increasing the number of WG arms. The proposed principle was also experimentally demonstrated in [143], in which data transmission using the proposed PIC was performed for two multiplexed OAM states. The annual grating is also used in [144], where the authors show a structure for producing and encoding OAM modes that includes a ring cavity, a bus WG, and eight download units that are evenly connected to the ring and spread evenly around the circle. The electrically controlled thermo-optic effect in [145] demonstrated the dynamic switching of nine OAM modes with azimuthal polarization by using a spatially isolated μ-ring cavity and scattering unit that could be tuned individually. Figure 7a shows an applied cobweb-like unit consisting of a bus WG and a μ-ring hollow with 16 downloading WGs and 16 gratings. The micrograph of the fabricated emitter with the resolution of 200 µm is presented in Figure 7b, and the scanning electron micrograph with the resolution of 5 µm in Figure 7c. As it could be seen, the total diameter of the ring is 400 µm.

Another widespread solution is to obtain light enclosed in whispering gallery modes (WGMs) with high OAM into free-space beams with the well-controlled extent of OAM using Si-integrated optical OV emitters and angular gratings. The angular grating’s working theory is like that of second-order gratings, which are generally used as input/output couplers in straight WGs, in which the directed wave is dispersed by the grating elements collectively and a substantial fraction of power is redirected in a specific direction, resulting in constructive interference [146]. WGMs with high OAM can be assisted by circular optical resonators such as μ-rings or μ-disks. The example of silicon nitride μ-disk OAM-resonator was demonstrated in [147], where it was combined with an aluminium nanorod antenna array shaped in periodic rings for generation of a polarization-pure single OAM beam. The proposed unit cell engineering could provide many more possibilities for vector OV beams implementation; however, this approach is not without the disadvantage of simultaneous generating high-order parasitic modes. In [148] a μ-ring is represented by an angular grating structure with a periodic modulation of refractive index in the azimuthal direction which is embedded into the WGM resonator. The wavefront of the radiated light from the μ-ring skew in the azimuthal direction turns into a helix if the WG with grating is bent to form a loop. Theoretical predictions that the emitted beams hold precisely specified and customized OAM were also confirmed by experimental characterization.

The OAM beam emitter constructed on a shallow-ridge silicon μ-ring is proposed in [149] where the azimuthally distributed gratings generate an OAM beam, and each grating element is a hole etched on top of the WG to scatter radially polarized light. The key parameters, i.e., the number of grating elements and the radiation efficiency of a single element, are optimized to enhance the OAM order purity for *l* up to ±4. A variable amplitude splitter and an OAM emitter based on a μ-ring cavity are presented in [62]. Another research [150] employs a silicon-on-insulator μ-ring with three superimposed gratings, each designed to emit a different OAM order, as a device enabling 3 × 3 optical switching. This approach utilizing the ring resonators with gratings provides good mode purity for OAM-multiplexing, however, the OAM-beams are generated already out of the chip plane in free space and cannot be used or processed at the chip any more.

**Figure 8:**
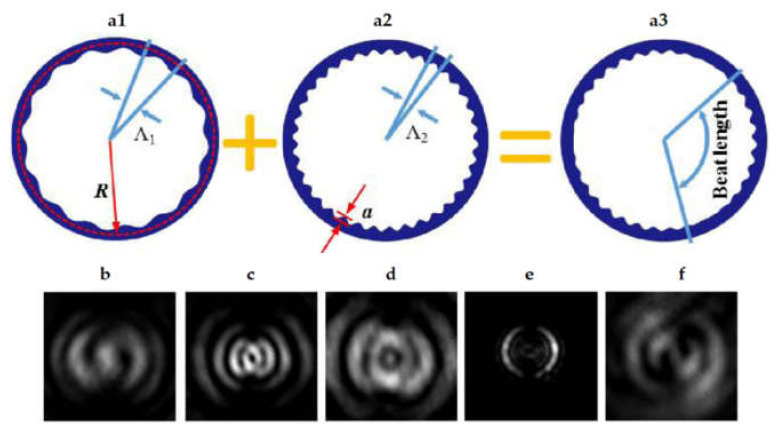
(**a1**,**a2**) Schematic of two single grating (with period of *Λ*_1_ and *Λ*_2_, respectively) superimposed into multiple-beat modulated device [151]. An unfolded view of resulting the three beats device (**a3**) [151]. The far-field patterns of the two-beat grating device: (**b**) 1508.1 nm, topological charges combination +1 and −1; (**c**) 1518.9 nm, topological charges combination 0 and −2; and (**d**) 1530 nm, topological charges combination −3 and −1 [151]. The near-field pattern is showing in (**e**), and the well-defined spiral interference fringe existed at appropriate locations is showing in (**f**) [151].

Finally, a generation of optical beams carrying superposition of OAM states by utilizing superimposed angular gratings embedded into WGM μ-ring resonators is described in [151]. Two sets of angular gratings with distinct period-*Λ*_1_ (Figure 8(a1)) and *Λ*_2_ (Figure 8(a2)) have been superimposed into a ring resonator in the device, resulting in a superimposed grating envelope with numerous beat perturbations, as illustrated in Figure 8(a3). Figure 8b–d shows far-field images of the two-beat grating device recorded at various resonant wavelengths according to the transmission/radiation spectrum, resulting in various topological charge combinations. The near-field pattern (Figure 8e) and the well-defined spiral interference fringes (Figure 8d) show two crescent-shaped lobes positioned in a circular pattern, showing that the radiated beams are truly emitted from the modulated gratings. Because it does not require any extra couplers or MZIs, the suggested device is quite small. Furthermore, by simply adding more gratings, it may construct superposition states in more than two dimensions. Because all modes are emitted from the same source mode via the same superimposed grating, the used methodology is simple and maintains a fairly constant phase between the OAM states.

**Figure 9:**
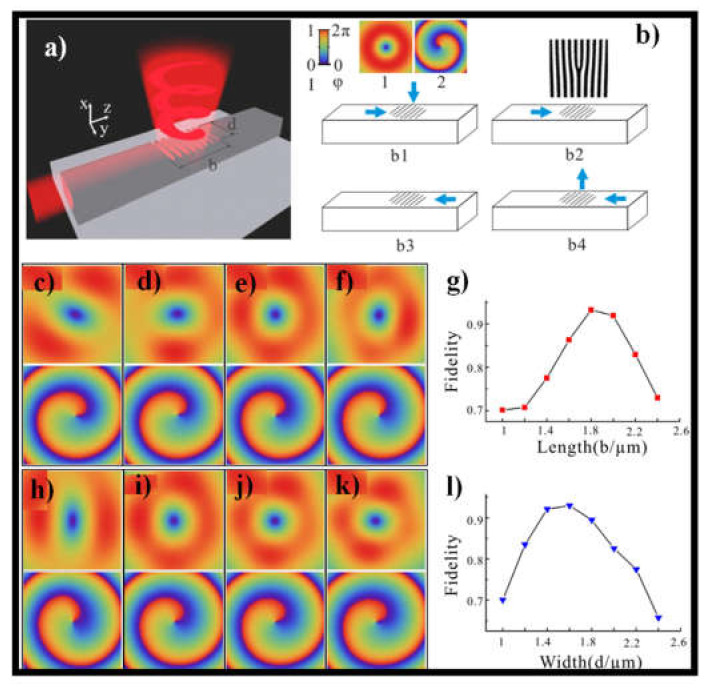
(**a**) The graphical image of the OV beam generator, (**b**) (**b1**–**b4**), the basis of the holographic grating on WG. The acquired OV beams by the holographic gratings with different sizes. The intensity and phase distributions are shown in the upper row and down row of the figures, (**c**–**f**) consistent to the holographic gratings with a constant width *d* = 1.5 μm but different lengths *b* = 1, 1.4, 1.8, 2.2 μm, and (**h**–**k**) corresponding to the holographic gratings with a constant-length *b* = 1.8 μm but dissimilar widths *d* = 1, 1.4, 1.8, and 2.2 μm, respectively, (**g**,**l**) are the fidelities of the attained OV beams as the functions of length *b* and width *d*, correspondingly. Reprinted with permission from ref. [152]. Copyright 2016, Elsevier.

Integrated WGs could be also applied in the out-of-plane approach. In [148] optical OV beam with a well-defined and continuously tunable OAM state is generated using the silicon bus WG that forms an open circle. A multi-WG surface holographic gratings composed of four WGs perpendicular to each other with holographic gratings etched on their surfaces is proposed in [61] for high-order OAM generation up to *l* = +8. In [152,153], the free space OV beam and the in-plane guiding wave can be transferred to each other by placing a holographic grating on top of a dielectric WG. The geometry parameters of this μ-scale holographic grating are extremely stable. Furthermore, multiple generators made up of two holographic gratings on two parallel WGs are investigated, which can modulate the OV beam in an efficient and versatile manner by regulating the phase of the input light. Figure 9a shows a schematic of the proposed OV beam generator, in which incident light from one WG port (in-plane) is transformed to free space (vertical) OV beam by the WG surface holographic grating (WGSHG).

The essential idea of holographic grating is shown in Figure 9(b1–b4). Figure 9c–f shows the simulation findings of the acquired beam for holographic gratings with a constant width *d* = 1.5 μm and different lengths *b* = 1 μm, 1.4 μm, 1.8 μm, and 2.2 μm. Figure 9g shows the OV beam fidelities as a function of length b, where the fidelities are larger than 0.7 for b in the range [1,2.4] μm. Fidelity is calculated by using the following formula:

$$F=\frac{{\left|\int A_{o}^{*}\left(x,y,z\right)A_{t}\left(x,y,z\right)dxdydz\right|}^{2}}{\int {\left|A_{o}\left(x,y,z\right)\right|}^{2}dxdydz\;\int {\left|A_{t}\left(x,y,z\right)\right|}^{2}dxdydz},$$

where *A_o_*(*x*,*y*,*z*) and *A_t_*(*x*,*y*,*z*) are the amplitudes of the obtained and target vortex beams, respectively [152]. Fidelity surges with *b* until it hits a peak, after which it drops with *b*. The greatest fidelity is 0.93 for *b* = 1.8 μm, which is the length of the holographic grating in the accompanying figure. The OV beam is formed based on the grating width d and the fixed length *b* = 1.8 μm, as shown in Figure 9h–l. The field distribution for *d* = 1 μm, 1.4 μm, 1.8 μm, and 2.2 μm indicate that d has a moderate effect on phase but a considerable effect on amplitude. The fidelity represented in Figure 9l has an optimal *F* = 0.93 for *d* = 1.6 μm.

The main disadvantage of the WGMs OAM emitters is that their inherent narrow bandwidths prevent them from being compatible with WDM/FDM techniques. To overcome the μ-ring’s bandwidth restriction, freeform MS was demonstrated in [154], utilizing a novel joint path-resonance phase control concept. The proposed SOI emitter is based on series of subwavelength cavities with low Q resonance, providing phase control and short response time for an ultra-broadband operation. [154] proposes an OV beam generation method based on elliptical nanoholes array designed on the MS with spiral phase distributions.

**Figure 10:**
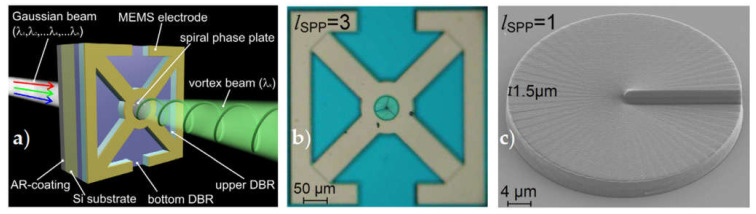
(**a**) Scheme of an OV MEMS Fabry-Perot filter: AR-coating = anti-reflection coating, and DBR = distributed Bragg reflector [160]. (**b**) Top view of a MEMS tunable Fabry-Perot filter with an integrated SPP [160]. (**c**) SEM image of a SPP of order *l*_SPP_ = 1 on a plane Si substrate [160].

OAM beams can also be generated directly by adding an integrative OV generator module to the light source’s output port or directly generating a OV beam within the light source cavities [155]. In this manner, integrated micro SPPs and two-dimensional fiber-gratings placed at the output port of a single-mode 860 nm vertical-cavity surface-emitting laser (VCSEL) were utilized in [65] and [156], respectively. This approach has shown robustness and high purity of the generated OAM states, allowing OAM multiplexed data transmission [157,158]. Fabrication of a broadly wavelength tunable MEMS-based Fabry-Perot filter with the sized SPP placed on the filter aperture has been proposed in the papers with our contribution [159,160]. This device divides wavelengths in a controllable manner while also giving a fixed OAM determined by the topological charge of SPP in the output beam. Figure 10a,b shows the construction and top view of a constructed OV emitter that included an anti-reflection coating, a Si substrate, a fixed bottom distributed Bragg reflector (DBR), a changeable air gap, a moveable MEMS DBR, and an SPP. Figure 10c shows a SEM image of the first-order SPP that will be inserted into the circular aperture in the Cr/Au layer of the MEMS DBR.

Notwithstanding that most of the proposed methods are experimentally verified, telecom applications are demonstrated only for ring resonators with annular grating [61,148,150], fixed SPPs [65,159,160], and lately for a pixel-array structure [154]. The output OAM beam from most out-of-plane generation methods is satisfactory in terms of mode purity; nevertheless, free-space propagation necessitates the use of a lens to concentrate the signal and couple it into the optical fiber, which reduces the final device’s reliability and scale. The minimal potential for multiplexing of OVs with different topological charges is another drawback of this method.

For more complex operations using vortices on a photonic integrated circuit, it is necessary to generate or inject a beam into the chip’s plane, and on-chip WGs must support its propagation. To implement such an operating principle, not only functional methods are important, but also the features of the chosen PIC platform. The most common integrated photonics platforms which are used for in-plane OAM applications are based on Silicon-on-Insulator (SOI) [161,162,163,164] and Silicon Nitride (SiN_x_) [165,166,167]. Because of the low cost, the high material strength of Si and CMOS compatibility, PICs based on the Si platform are particularly well suited as a medium for integrating with other components. Moreover, in [153,168] a scheme is proposed that uses a hybrid plasmonic WG to produce light beams with selective angular momentum (AM) over a wide wavelength range. AM beams can either propagate through optical WGs or emit into free space. Since it is based on an SOI platform and uses standard CMOS technology, this approach has high fabrication feasibility.

Regarding the propagation, it is experimentally demonstrated in [169,170] that OAM mode transmit over a rectangular polymer WG. Experimental data show that the imperfect shape of the intensity profiles and phase fronts of OV beams is caused not by the distortion of the OV modes propagating along the WG, but by the imperfection of the WG end facet, which affects the measured interferograms. This work provides the prototype illustration that OAM modes can propagate through rectangular on-chip WGs, proficient of traditional lithographic microfabrication.

**Figure 11:**
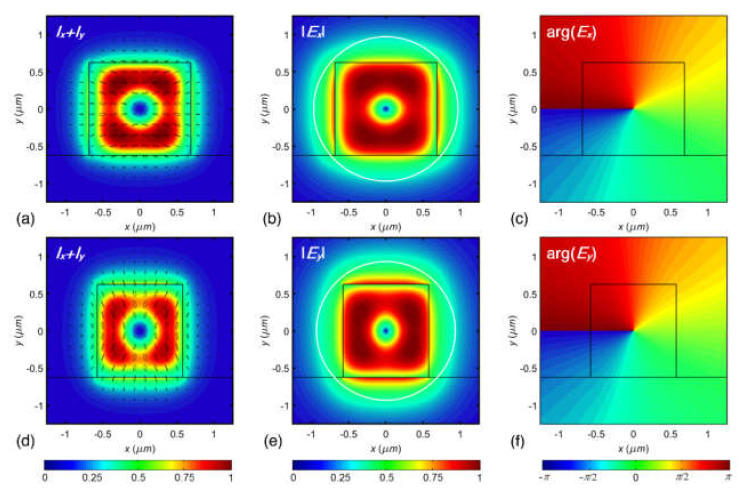
The normalized transversal field component intensity distribution superimposed with polarization map (**a**,**d**) [166], Normalized absolute amplitude mapping of dominant E-field component (**b**,**e**) and phase distribution of dominant E-field component (**c**,**f**) of the quasi-TE (**a**–**c**) and quasi-TM (**d**–**f**) quasi-degenerate modes of order *l* = 1 in the symmetric (silica clad) silicon nitride WGs enhanced for phase-matched propagation of the constituent eigenmodes (β_01_ ≈ β_10_) [166].

In our recent work [166] we conducted a computational and theoretical investigation into the prospect of OV modes transmission over dielectric rectangular WGs, as well as the issue of WG structure enhancement for the sustenance of OV modes. The findings show that rectangular WGs can theoretically transmit quasi-TE and quasi-TM modes with high OAM purity states in the dominant field component. The constituent eigenmodes of higher azimuthal orders can only propagate in a phase-matched regime for the quasi-generate mode of azimuthal order ±1, and the OV modes of higher azimuthal orders can only transmit with a particular beat length. Furthermore, the normalized power of the subsequent OAM state in the modal superposition declines as the target azimuthal order increases. Numerical EM simulations of silicon nitride WGs provided field mappings and OAM spectra of the related modal superpositions, confirming the analytical predictions. The normalized intensity distribution, normalized amplitude distribution, and phase mapping of the leading E-field component are shown in Figure 11a–f [166].

**Figure 12:**
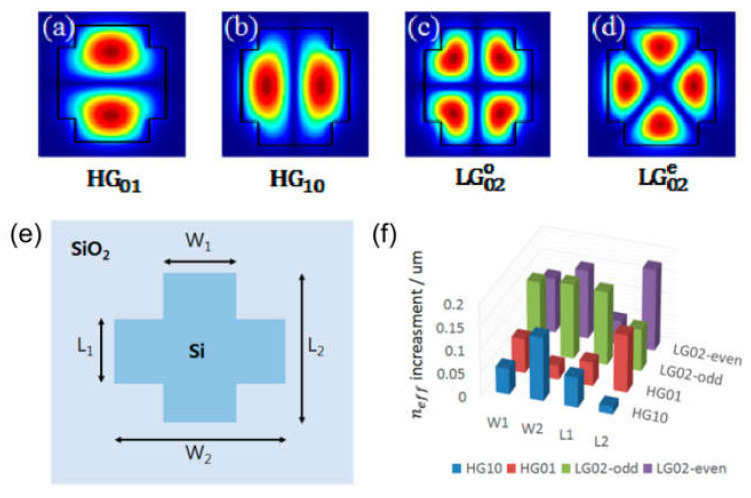
HG-similar mode field allocations in the WG (**a**–**d**) [163], WG configuration for concurrently managing *l* = ±1 OAM mode and *l* = ±2 OAM mode (**e**) [163], mode *n_eff_* reliance on WG parameters (**f**) [163].

In [163] SAM and OAM of light in silicon channel WGs was studied, where OV beam carrying OAM represented by a superposition of the TE_01_ and TE_10_ modes. Due to the transverse confinement, the SAM and OAM of the OV fields are strongly coupled and the whole space of structure variables of the WG can be separated just into three regimes. These results shed light on the correlation between angular momentum and mode confinement, which is useful for applying OVs in PICs. In the case of higher-order OAM modes propagation, the cross-shaped core WG structure was designed in [161]. Two degenerate driven modes of π/2 *l*-rotation symmetry can assist the *l*-th order OAM mode. The designed cross-shaped WG supports OAM modes of ±1 and ±2 topological charges concurrently at a wavelength of 1550 nm with high mode purity. The Hermite Gaussian (HG)-similar guided modes are shown in Figure 12a–d. To fulfil the degeneracy of HG_01_–HG_10_ and ${\mathrm{LG}}_{02}^{e}–{\mathrm{LG}}_{02}^{o}$, which form OAM modes of ±1 and ±2 topological charges, respectively, the geometric variables denoted as W_1_, L_1_, W_2_ and L_2_ have been optimized (see Figure 12e). The impact of every parameter on n_eff_ of the modes is shown in Figure 12f. Additionally, such WG structures were designed for *l* = ±3 and *l* = ±4 OAM modes guiding individually, but the purity turned out to be lower and it is challenging to design the WG concurrently supporting the *l* = ±3 and *l* = ±4 OAM modes. Probably, a more complex transverse composition is required to further increase the topological charge. Anyway, in-plane operation using higher-order OAM modes is a competitive field.

**Figure 13:**
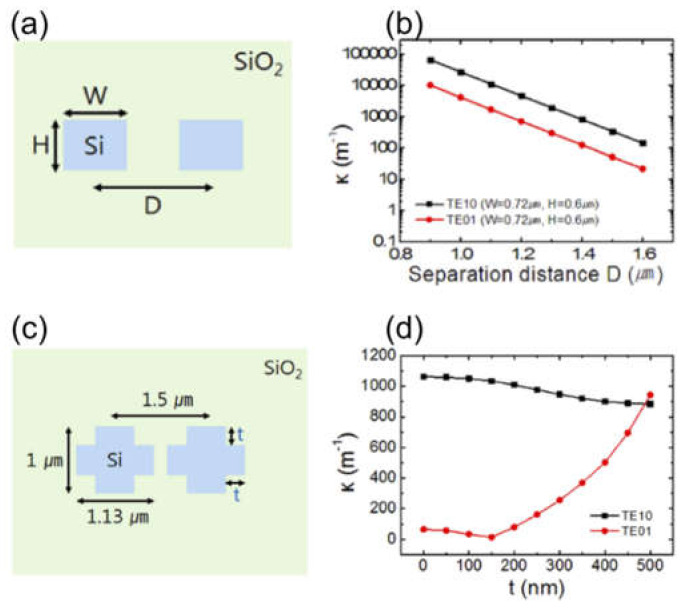
Cross-section of rectangular WG coupler (**a**) [164], the coupling coefficient of TE_10_ and TE_01_ modes in case of W = 0.72 µm and H = 0.6 µm (**b**) [164], the cross-section of cross shape WG coupler (**c**) [164], the coupling coefficient of TE_10_ and TE_01_ modes as a function of t (**d**) [164].

Furthermore, the same group discovered that an OAM mode directional coupler with fabricable dimensions might be difficult to achieve with a rectangular WG and presented an on-chip device based on the cross-shaped WG structure [164]. The structure is effective in balancing the horizontal direction coupling strength of OAM*_l_*_=1_ mode’s two constitutive eigenmodes. The desired device has a coupling length of 670 μm and exhibits little OAM mode purity loss during the optical power transfer procedure. But again, the design of such a device for the higher-order OAM modes coupling is tricky and still competitive. Figure 13 shows the dependences of the coupling coefficient on the length in the case of the usual directional coupler and on the cutout size t in the case of cross shape WG coupler. As can be seen, the coupling coefficient of the TE_10_ mode is always higher than the TE_01_ one in the case of a rectangular WG coupler and does not satisfy a requirement for the OAM*_l_*_=1_ mode directional coupler. But when the directional coupler is made of a cross-shaped WG, there is a cutout size around 490 µm, where TE_01_ and TE_10_ modes have the same coupling coefficient. The insertion of the cutoff breaks down the degeneracy between TE_10_ and TE_01_ modes in the starting rectangular WG. The particle swarm optimization method can be used to optimize WG’s width and height and the cutoff dimensions of the cross-shaped WG simultaneously [164].

To achieve a compact full-fledged device utilizing in-plane OV beams it is necessary to generate such carrying OAM state wave on an integrated circuit. In [161], it is proposed to produce OAM beams by a simple on-chip integrated structure involving only silicon WGs and couplers, which controls the phase shift of the second-order propagating mode. The idea of getting the necessary distribution at the output of the device already touched upon TE_01_ and TE_10_ eigenmodes being mixed with π/2 phase shift. The rectangular WGs assisting multiple transverse modes and keeping symmetry in two orthogonal transverse directions are also proposed in [171].

Another generation method—based on a hybrid plasmonic WG—is proposed in [153,168,172,173]. The main feature of the suggested method is to divide a WG into three sections: the first and third sections are pure dielectric WGs, and the middle section is a hybrid plasmonic WG. Because of the polarization rotation in the asymmetrical WG, the transverse field factors produce a spin angular momentum (SAM). The proposed method for generating an OAM beam in a WG could be useful for on-chip integrated optical tweezers, data processing, and other applications [153]. Later, the application of integrable quarter-wave plates being able to control the OAM of photons within nanophotonic WGs was proposed [174]. Although plates do not act as the generating structure, the fundamental physics embraces fundamental potential and applicability to an extensive variety of applications, such as detection of chiral molecules, nanoscale optical manipulation, integrated computing science, and on-chip managing of quantum information [174].

**Figure 14:**
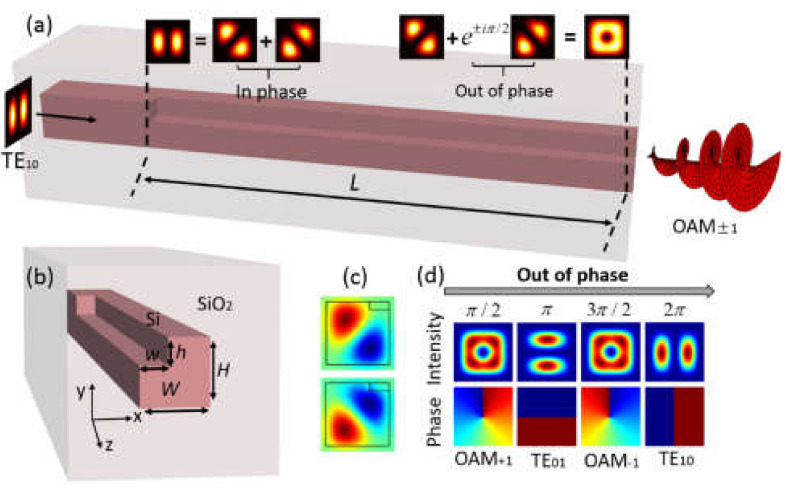
(**a**) OAM beam generator principle built on a single-trench WG; (**b**) A single trench WG in cross-section; (**c**) Two eigenmodes of a single-trench WG’s field distributions; and (**d**) For *x*-polarization, intensity and phase evolutions of a combination of eigenmodes [67].

Finally, an ultra-compact integrated structure to generate OAM modes is proposed by a specially designed single-trench WG in [67]. To implement OAM modes generation, the WG with a trench is designed. A trench can break the original rotation symmetry and split the mode degeneracy. As can be seen from Figure 14a, OAM modes with topological charge ±1 synthesized from two orthogonal LP-like eigenmodes that were excited by second-order TE_01_ mode at the input of a trench WG. Figure 14b shows a cross-section of a WG. The distributions of eigenmodes of a trench WG are shown in Figure 14c and its combination evolutions are presented in Figure 14d. Moreover, the structure of an on-chip OAM modes (de)multiplexer for *x*-polarization, utilizing the trench WGs was demonstrated.

Another method involves coupling incident radiation into an integral WG through an MS [167]. A Si antenna array on the surface of the Si_3_N_4_ WG represents the MS. MS simultaneously realizes OAM excitation and light coupling. The device also generates and adds two high-order TE_01_ and TE_10_ modes with π/2 phase difference. The convenience of the MS in simultaneous excitation and mixing of modes at the required phase ratio.

Finally, the authors have recently introduced the principle of vortex mode generation by coherent superposition between two high-ordered TE-like modes excited in one waveguide from the incident radiation using the grating coupler. First, in [175] the approach was demonstrated based on the two waveguides of different widths, which are coupled together by the directional coupler. Next, in [176] the symmetric silica-cladded silicon nitride WG with shallowly etched gratings is provided for the in-plane generation of the vortex mode with azimuthal order *l* = ±1. The proposed design is beneficial over the previous because it provides the generation of a pure phase vortex of quasi-TE polarization at the PIC output; the device can be implemented as a passive PIC and can be excited by a single standard SMF, which can be coupled in a technologically convenient in-line way. The power coefficients of the target OAM states ±1 are higher than 0.96, which is verified with the numerical simulation. The only drawback is relatively low coupling efficiency compared to the typical values for fundamental TE00 mode excitation, which however could be further increased by properly optimizing the etching depth and other grating coupler parameters.

## 5. Detection of Light with OAM

OAM detection at the receiver side is needed in communications. Even though OAM detection is simply the reverse of OAM generation, it is significantly difficult because of the deteriorated OAM states after transmission [177]. Mode assessment established on field data [178], the study of OAM induced effects—for instance, the rotational Doppler shift [179,180]—and beam-restoring using holographic technology [181] are the three types of OAM detection. To measure the OAM directly, the first method requires acquiring all three components of E and H-fields [182]. On the other hand, the phase gradient equal to the OAM index *l* can be calculated. The OAM modes can be anticipated into a measurable Gaussian mode in the third method, which utilizes a hologram [183]. Geometric-phase MSs can be employed to integrate holograms because they have a low profile and a lot of tuning versatility.

**Figure 15:**
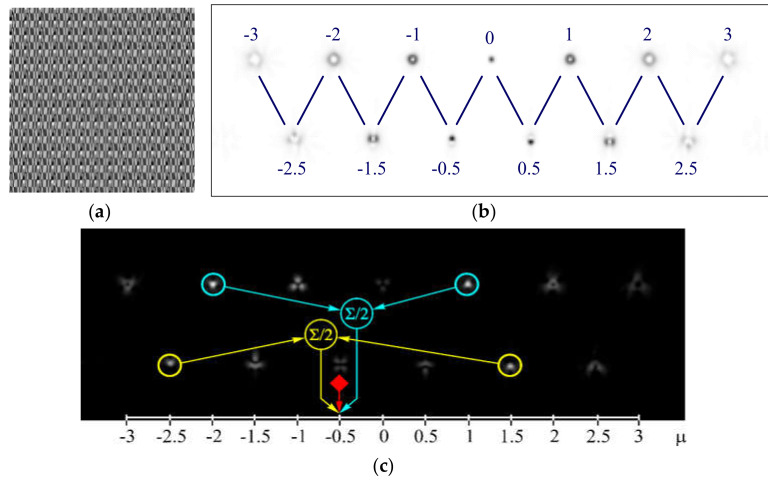
Detecting OVs: (**a**) phase of a 13-channel filter; (**b**) intensity pattern (negative) in the focal plane and correspondence of diffraction orders to OV values; and (**c**) results of detecting of different OVs in the beam with co-axial superposition exp(−*i*2φ) + exp(−*i*φ) (total OAM μ = −0.5) [194].

Based on DOEs, multi-channel (or multi-order) optical elements have been developed, matched to OVs of different orders [184,185,186,187] and their action is based on the correlation filtering in the Fourier region [188]. Subsequently, these DOEs were used in the problems of fiber-optic multichannel communication utilizing OV beams [4]. More recent studies used SLMs [189,190,191,192,193]. For example, Figure 15 shows detecting results for the phase 13-channel filter, matched with integer and half-integer OVs [193]. Note, DOEs can be employed to recognize not only the singular phase state of structured light beams but also for detection polarization state [4,5,6,194]. However, MSs are more suitable for these purposes [195,196,197,198,199,200].

The additional degree of freedom provided by the OAM state of photons has a wide range of uses. For enhanced fidelity, miniaturization, and reconfiguration of OAM state measurement, which is a fundamental requirement of these applications, photonic integrated devices are required. In [201], a Si-integrated OAM receiver is described that can discriminate between separate and changeable OAM states. Also, the detector’s reconfiguration capability is attained by using a voltage to the GeSe film, which forms gratings with alternate states. By modifying the duty cycle of the gratings, the resonant wavelength for any OAM state is shown to be tunable in a quasi-linear manner. It proposes a viable method for realizing a compact integrated OAM detection system with better functionality, which could have vital applications in optical communications and information processing involving OAM states [199]. Photonic integrated circuits (PICs) perform a wide range of optical functions using light rather than electrons. The spectrum of potential functionalities for these highly integrated optical chips has been extended thanks to recent advances in nanostructures, metamaterials, and Si technologies. Mach–Zehnder interferometric and ring-based modulators, for example, have been published [202,203,204,205,206,207]. Work on an OAM detection system, which includes converting light with distinct OAM states into spatially separated surface plasmon polaritons waves, has begun in recent years. Miniature photonic devices with dimensions significantly smaller than those now accessible are possible thanks to the surface plasmon polariton’s unique features in terms of short equivalent wavelength and excellent spatial confinement. Metaholograms with plasmonic photodiodes, for instance, have been shown to detect OAM in real time [180]. This method allows for the development of a compact, integrated detection system. It is, however, limited to one OAM state. Several methods for the detection of OAM of light has been proposed in recent times [208,209,210,211].

**Figure 16:**
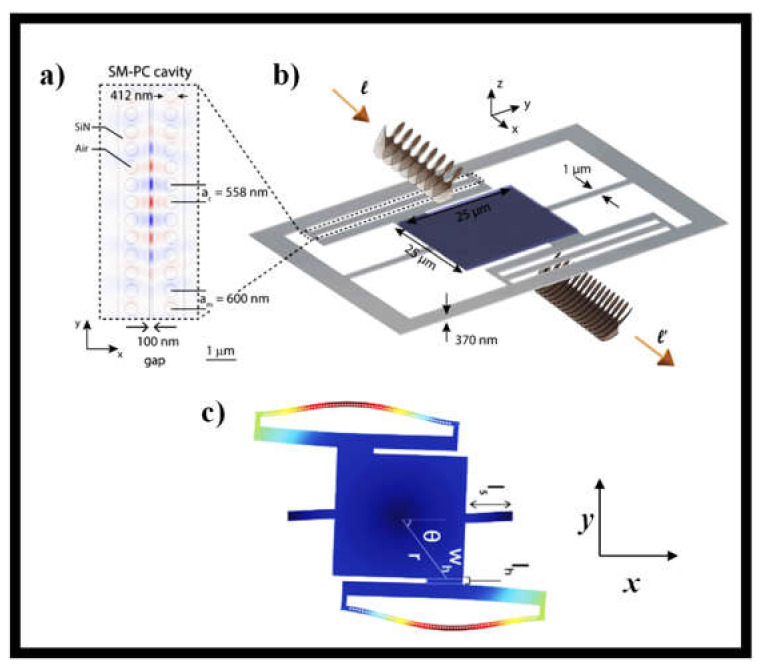
A diagram of the device’s geometry: (**a**) Top view of a slot-mode PhC cavity geometry and modeling of the E-field distribution of its fundamental optical mode [212]; and (**b**) The OAM detector in isometric perspective. The cavity from (**a**) is attached to the square pad by a hanger whose dimensions *w_h_* and *l_h_* are indicated in (**c**) [212]. The pad motion is moved to the nanobeam when excited by a source of torque as exhibited by the simulated displacement profile shown in (**c**) [212].

A novel concept of an optomechanical system that enables sensitive transduction of OAM of light has recently been demonstrated [212]. A photonic crystal cavity optomechanical system detects an optically induced twist on the chip. This facilitates the measurement of the OAM of light as photons are absorbed by the mechanical element or detect photons as they are dispersed into new OAM states by a sub-wavelength grating patterned on the device. Such a device will detect optical pulses with *l* = 1 OAM field and an average photon number of 3.9 × 10^3^ at a repetition rate of 5 MHz, assuming detector noise does not restrict measurement precision. High-order OAM states can be added to this design. A schematic of the OAM detector system reported in [212] is shown in Figure 16. As shown in Figure 16a,b, it comprises of a center suspended pad connected to one side of a slot-mode photonic crystal nanobeam cavity [212].

The chip that surrounds the cavity is linked to the other half of the cavity. The cavity operates in an air-band mode with a field centered in the space between the nanobeams, making it very sensitive to any nanobeam motion that changes the slot-gap width, causing the cavity mode wavelength to change. The motion of the nanobeam is transformed into the amplitude of light coupled out of the cavity during this dispersive optomechanical coupling phase. Light may be linked into and out of the cavity using an optical fiber taper WG evanescently interfering with the cavity to control this optomechanical transduction. By rotating the central pad and activating the nanobeam’s mechanical bounce mode, optical actuation of the central pad through OAM moves the nanobeam’s center of mass location. Figure 16c shows a simulated displacement profile of the unit when its central pad is operated by an external torque source, displaying several forms of displacement [212]. The OAM of light incident on the device’s central pad may be transformed to torque using a variety of different approaches. The OAM of light changes from *l* = *l*′ = 0 as the torsional pad is covered with an absorbent layer and lit up with helical light. A helical beam with OAM number *l* can be altered during broadcast or reflection to any OAM number *l*′ supplied by the MS shape if the torsional pad is replicated with an appropriately built MS.

## 6. Conclusions

In this paper, we have reviewed the most extensively used methods for the generation of orbital angular momentum (OAM) optical beams and their detection. These methods include the usage of diffractive optics, metasurfaces (MSs), and photonic integrated circuits (PICs). Diffractive optics can be further classified in spiral phase plates, computer-generated holograms, and diffractive optical elements. MSs are relatively compact and deliver high performance compared to conventional DOEs. PICs based OAM generators can be divided into two sub-categories: out-of-plane OAM generator and in-plane OAM generator. For the latter, it has attracted a lot of interest in fully utilizing the unique feature of OAM beams in the guided optics. However, to ensure uniform spatial distributions of the multi-coupled waveguide, the majority of the integrated OAM generators need exceedingly critical dimension control technology. The aim of this paper is not to highlight the dominance of one approach over another. We have provided the advantages/disadvantages of each method based on the ease of implementation and performance.

## Data Availability

Not applicable.
